# Role of the endocannabinoid system in fragile X syndrome: potential mechanisms for benefit from cannabidiol treatment

**DOI:** 10.1186/s11689-023-09475-z

**Published:** 2023-01-09

**Authors:** Joseph M. Palumbo, Brian F. Thomas, Dejan Budimirovic, Steven Siegel, Flora Tassone, Randi Hagerman, Christopher Faulk, Stephen O’Quinn, Terri Sebree

**Affiliations:** 1 Zynerba Pharmaceuticals Inc., Devon, PA USA; 2 Empirical Pharmaceutical Services, LLC, Manteo, NC USA; 3 Departments of Psychiatry and Neurogenetics, Fragile X Clinic, Kennedy Krieger Institute, Baltimore, MD USA; 4 Department of Psychiatry & Behavioral Sciences-Child Psychiatry, Johns Hopkins School of Medicine, Baltimore, MD USA; 5 Department of Psychiatry and Behavioral Sciences, Keck School of Medicine, University of Southern California, Los Angeles, CA USA; 6 Medical Investigation of Neurodevelopmental Disorders (MIND) Institute, University of California-Davis Medical Center, Sacramento, CA USA; 7 Department of Biochemistry and Molecular Medicine, School of Medicine, University of California-Davis, Sacramento, CA USA; 8 Department of Pediatrics, University of California Davis School of Medicine, Sacramento, CA USA; 9 Department of Animal Science, University of Minnesota, St. Paul, MN USA

**Keywords:** Fragile X syndrome, Endocannabinoid system, Cannabinoid receptors, Cannabidiol

## Abstract

Multiple lines of evidence suggest a central role for the endocannabinoid system (ECS) in the neuronal development and cognitive function and in the pathogenesis of fragile X syndrome (FXS). This review describes the ECS, its role in the central nervous system, how it is dysregulated in FXS, and the potential role of cannabidiol as a treatment for FXS. FXS is caused by deficiency or absence of the fragile X messenger ribonucleoprotein 1 (*FMR1*) protein, FMRP, typically due to the presence of >200 cytosine, guanine, guanine sequence repeats leading to methylation of the *FMR1* gene promoter. The absence of FMRP, following *FMR1* gene-silencing, disrupts ECS signaling, which has been implicated in FXS pathogenesis. The ECS facilitates synaptic homeostasis and plasticity through the cannabinoid receptor 1, CB_1_, on presynaptic terminals, resulting in feedback inhibition of neuronal signaling. ECS-mediated feedback inhibition and synaptic plasticity are thought to be disrupted in FXS, leading to overstimulation, desensitization, and internalization of presynaptic CB_1_ receptors. Cannabidiol may help restore synaptic homeostasis by acting as a negative allosteric modulator of CB_1_, thereby attenuating the receptor overstimulation, desensitization, and internalization. Moreover, cannabidiol affects DNA methylation, serotonin 5HT_1A_ signal transduction, gamma-aminobutyric acid receptor signaling, and dopamine D_2_ and D_3_ receptor signaling, which may contribute to beneficial effects in patients with FXS. Consistent with these proposed mechanisms of action of cannabidiol in FXS, in the CONNECT-FX trial the transdermal cannabidiol gel, ZYN002, was associated with improvements in measures of social avoidance, irritability, and social interaction, particularly in patients who are most affected, showing ≥90% methylation of the *FMR1* gene.

## Introduction

### Fragile X syndrome

Fragile X syndrome (FXS) is a neurodevelopmental genetic disorder that has a prevalence of approximately 1 in 4000 males and 1 in 6000 females [1]. The main genetic mutation that causes FXS is a trinucleotide repeat expansion of the sequence cytosine, guanine, guanine (CGG), with 200 or more repeats in the 5’ untranslated promoter region of the fragile X messenger ribonucleoprotein 1 (*FMR1*) gene (>200 repeats represents full mutation [FM]), which encodes the FMRP protein and is located on the X chromosome [2, 3]. FM leads to epigenetic methylation of the gene and consequent absence of *FMR1* mRNA transcription and translation of FMRP [2, 4–6]. Thus, FXS is caused by the deficit or absence of FMRP [7], an RNA-binding protein important for normal synaptic function, synaptic plasticity, and for the development of neuronal connections over time during brain maturation [8].

FXS is associated with a wide range of neurobehavioral impairments in skills (i.e., cognitive, language) and behaviors, including autism spectrum disorder (ASD), anxiety, aggression toward others, irritability, temper tantrums, shyness, and preference for solitary activities [9–11]. In general, the FXS neurocognitive and behavioral phenotype depends on the amount of FMRP that is produced, which is determined in part by the degree of the methylation of *FMR1* [12, 13]. Males with the FM and full methylation generally do not produce FMRP, whereas in females with the FM and full methylation the protein can range from near normal to significantly reduced expression of FMRP, depending on the pattern of X-inactivation in the affected female [14, 15]. In general, patients with FXS with a higher degree of methylation have a more severe phenotype such as lower IQ, and may have more severe symptoms of ASD, although there is wide variability for any given level of methylation [12, 15–17]. Individuals with a high degree of mosaicism due to the presence of cells carrying FM alleles and cells carrying alleles in the premutation range (i.e., 55 to 200 CGG repeats) or unmethylated FM alleles may produce elevated *FMR1* mRNA, which in itself can cause RNA toxicity to the cells of the central nervous system (CNS) [18, 19]. Those with FM and full methylation of *FMR1* produce reduced amounts of *FMR1* mRNA and little to no FMRP [12]. Therefore, they resemble the classical and most severe phenotype of FXS, characterized by lack of FMRP, which is recapitulated by the knockout mouse model of FXS [20]. Despite decades of preclinical research and interventional clinical trials, no approved treatments exist for FXS [21].

### Purpose of this review

Multiple lines of evidence suggest a central role for the endocannabinoid system (ECS) in the neuronal development and cognitive function and the pathogenesis of FXS. This review describes the ECS, its role in the CNS, how it is dysregulated in FXS, and the potential role of cannabidiol as a treatment for FXS.

### Role of the ECS in the CNS

The ECS is postulated to play a role in neuronal development and function, including facilitating synaptic homeostasis and plasticity [22]. The ECS primarily includes the endocannabinoids, 2-arachidonoylglycerol (2-AG) and anandamide (AEA), and the cannabinoid G-protein-coupled receptors, cannabinoid receptor 1 (CB_1_) and cannabinoid receptor 2 (CB_2_) [23, 24]. CB_1_ and CB_2_ are selectively expressed in various tissues [23, 24]. CB_1_ receptors are expressed in the brain and are present at lower concentrations in a variety of peripheral tissues and cells. Brain regions that possess high levels of CB_1_ receptors include the neocortex, cerebellum, and forebrain structures, as well as the basal ganglia and limbic system areas that contribute to learning and memory, executive functioning, social interaction, and behavior and emotion. CB_2_ receptors are expressed primarily in the immune and hematopoietic systems, as well as in the brain, pancreas, and bone.

**Fig. 1:**
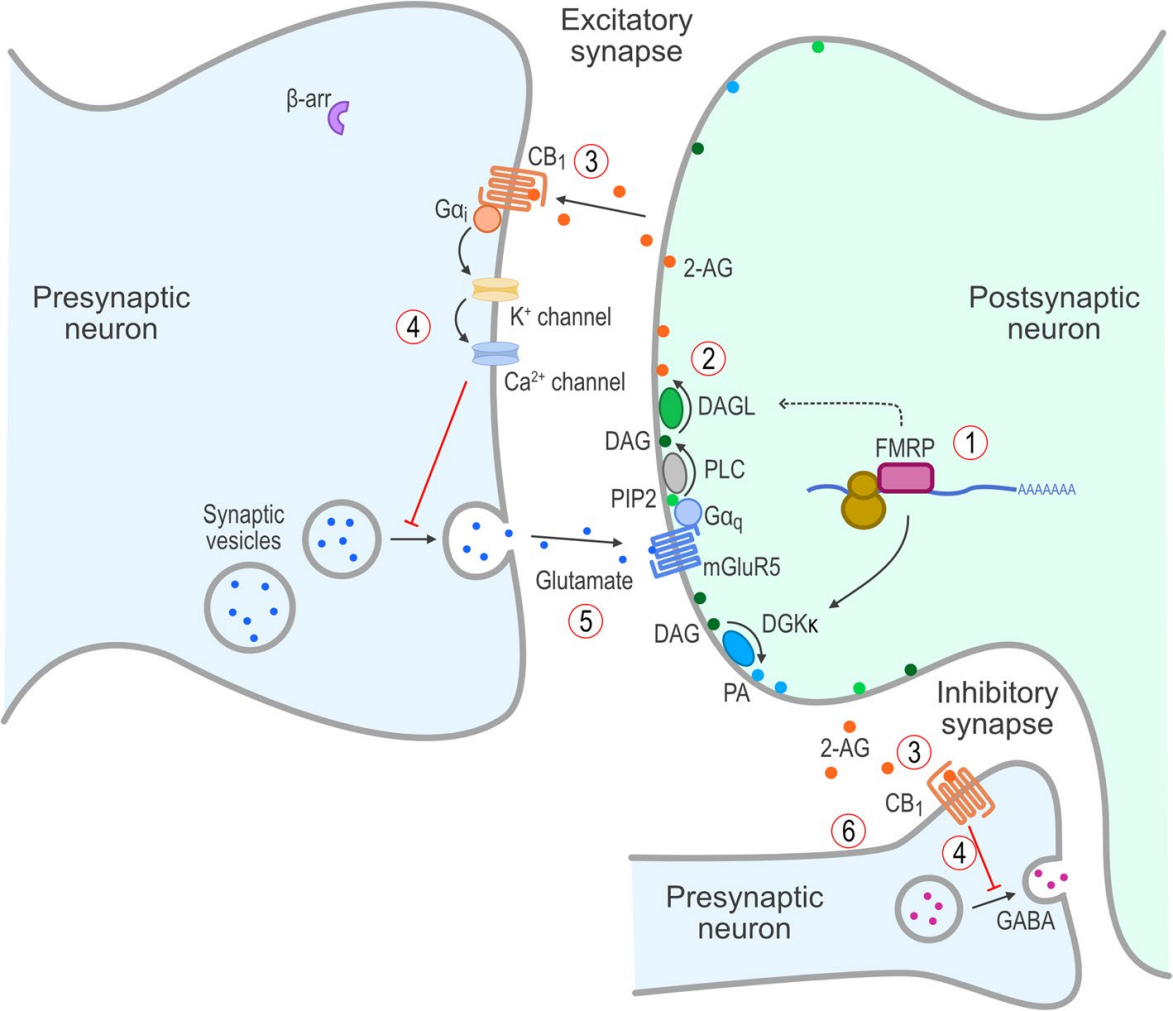
Endocannabinoid-mediated signaling in the CNS in the normal state. In a normal state with *FMR1* protein present, (1) FMRP supports expression of DGKκ and traffics DAGL mRNA, which results in (2) normal production of 2-AG and release into the synaptic cleft, which (3) stimulates presynaptic CB_1_ receptors resulting in (4) retrograde inhibitory signaling and (5) optimal release of glutamate and activation of mGluR5 receptors and (6) modulation of GABAergic function. 2-AG, 2-arachidonoylglycerol; β-arr, β-arrestin; CB1, cannabinoid type 1 receptor; CNS, central nervous system; DAG, diacylglycerol; DAGL, diacylglycerol lipase; DGKκ, diacylglycerol kinase-κ; FMRP, *FMR1* protein; G, G proteins; GABA, γ-aminobutyric acid; mGluR5, group I metabotropic glutamate receptor 5; mRNA, messenger RNA; PA, phosphatidic acid; PIP2, phosphatidylinositol-4,5-bisphosphate; PLC, phospholipase C

In the brain, endocannabinoids are synthesized and released “on demand” from postsynaptic membrane-bound phospholipids in response to neuronal signaling and act as retrograde signaling molecules across the synaptic cleft to stimulate CB_1_ receptors on the presynaptic terminal (Fig. 1) [23, 25] and attenuate further activity through an inhibitory feedback loop. Enzymes that function in synthesizing 2-AG include phospholipase C, diacylglycerol kinase-κ (DGKκ), and diacylglycerol lipase (DAGL) [23, 26]. At developed synapses, 2-AG released from postsynaptic terminals binds to presynaptic CB_1_ receptors to inhibit the secretion of both excitatory and inhibitory neurotransmitters [27]. As mentioned above, the elements that comprise the ECS (i.e., the endocannabinoids and their receptors, CB_1_ and CB_2_) are located in the CNS [28–30]. Evidence indicates that the ECS has an important role in the CNS and alterations in the ECS in experimental animal models results in profound changes in cognition and behavior [31, 32]. Thus, as the ECS appears to regulate neuronal development and function, particularly synaptic homeostasis and plasticity [22], pharmacological intervention of this pathway, when disrupted, could prove to be a beneficial approach for the treatment of cognitive and behavioral problems. Consistent with this hypothesis, several drugs that target the ECS are undergoing clinical development for neurodevelopmental and neuropsychiatric disorders [33–35].

### Dysregulation of the ECS in FXS

**Fig. 2:**
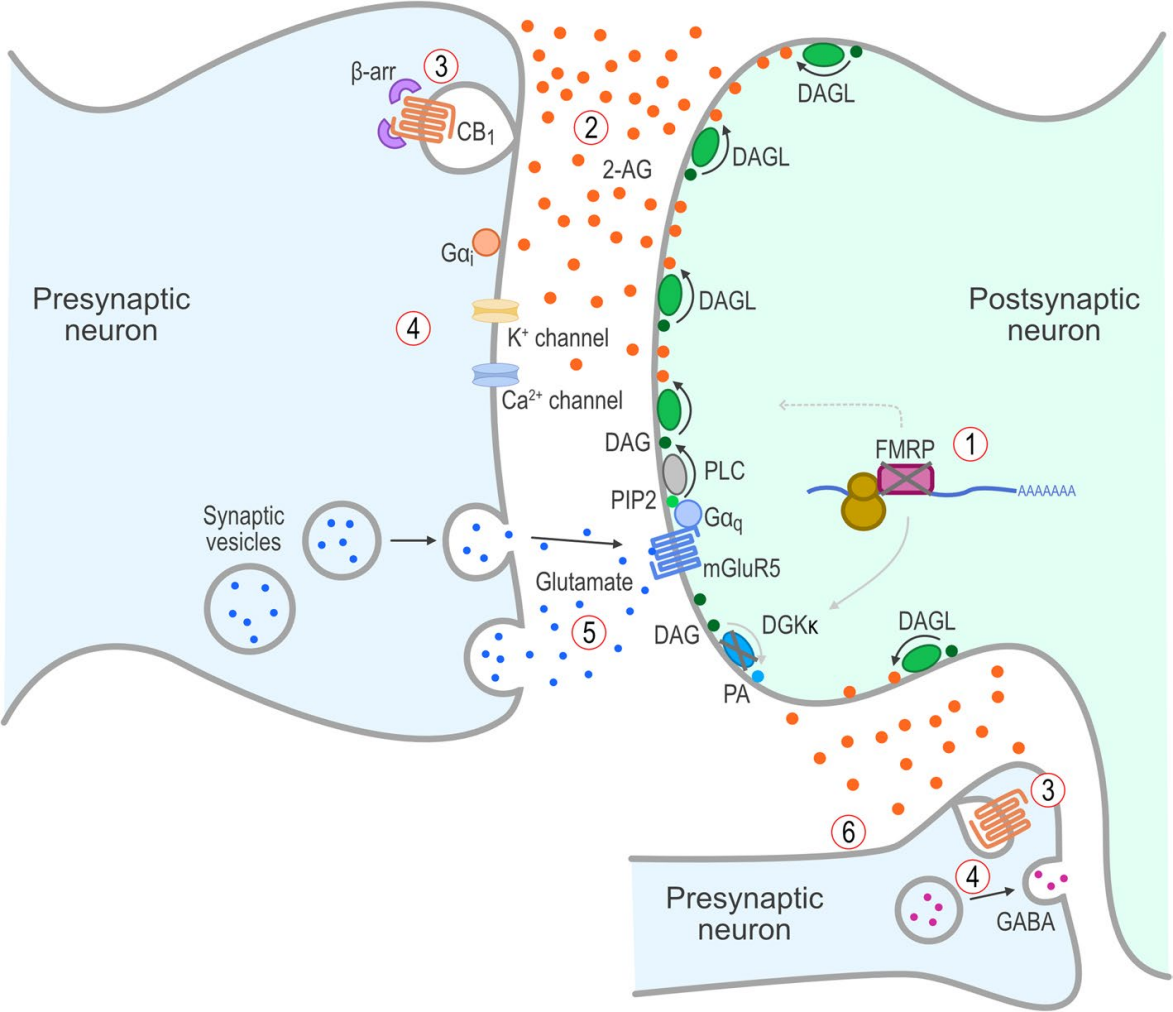
ECS dysfunction in FXS due to lack of FMRP. Lack of FMRP in FXS leads to (1) reduced expression of DGKκ and abnormal trafficking of DAGL mRNA, which results in (2) ectopic/abnormal production of 2-AG and release into the synaptic cleft, which causes (3) β-arrestin recruitment, internalization, and desensitization of CB_1_ receptors, resulting in (4) loss of the normal retrograde inhibitory signaling and (5) increased glutamate release and activation of mGluR5 receptors and (6) altered GABA release. 2-AG, 2-arachidonoylglycerol; β-arr, β-arrestin; CB1, cannabinoid type 1 receptor; CNS, central nervous system; DAG, diacylglycerol; DAGL, diacylglycerol lipase; DGKκ, diacylglycerol kinase-κ; ECS, endocannabinoid system; FMRP, *FMR1* protein; FXS, fragile X syndrome; G, G proteins; GABA, γ-aminobutyric acid; mGluR5, group I metabotropic glutamate receptor 5; mRNA, messenger RNA; PA, phosphatidic acid; PIP2, phosphatidylinositol-4,5-bisphosphate; PLC, phospholipase C

The functional consequences of significantly reduced or absent FMRP in people with FXS likely reflect changes in both developmental and dynamic regulation of multiple intracellular processes involved in controlling the structure and function of the synapses within the CNS. FMRP is a critical element of translational control in dendritic polyribosomes that has been implicated in the repression of mRNA translation during trafficking to dendrites and synapses [36]. Aberrant synaptic protein synthesis due to alterations in FMRP levels has been proposed as a possible pathway leading to autistic phenotypes [37]. With respect to the ECS, FMRP has a recognition motif for DAGL mRNA [27]. When FMRP is translated and binds to DAGL mRNA in the polyribosome, it acts as a translational repressor while it traffics the mRNA to the post-synaptic dendritic terminal. It has been suggested that decreased or absent FMRP disrupts normal DAGL trafficking and the formation of functional postsynaptic group I metabotropic glutamate receptor 5 (mGluR5)-DAGL complexes and disables on-demand endocannabinoid release and retrograde signaling in FXS, leading to ectopic production of 2-AG [27]. The resulting overstimulation of presynaptic CB_1_ receptors then causes β-arrestin recruitment and phosphorylation, internalization, and desensitization of CB_1_ receptors, and the dysregulation of retrograde endocannabinoid signaling in response to neuronal activity [38]. Therefore, absence of FMRP dysregulates the “on-demand” release of 2-AG via DAGL, thereby disrupting normal ECS function in feedback inhibition and synaptic plasticity (Fig. 2) [27, 39]. The loss of synaptic plasticity may result in deficits in learning, memory, and behavioral and emotional responsivity observed in FXS and other behavioral disorders [27, 40]. Specifically, reductions of FMRP are thought to impair ECS-mediated regulation of glutamate signaling and gamma-aminobutyric acid (GABA)ergic signaling in FXS [27, 39, 41]. Likewise, reductions in FMRP have been associated with altered ECS-mediated responses at GABAergic synapses [39, 41], suggesting disruption of retrograde signaling by the ECS at inhibitory synapses involved in GABAergic function in FXS. This disruption in ECS-mediated negative feedback of neuronal signaling may represent one of the key physiologic mechanisms underlying both the development of FXS neuronal dysfunctions and the expression of more debilitating behavioral symptoms, including severe social anxiety and irritability.

There is considerable preclinical and clinical evidence to support a link between the ECS and FXS and ASD phenotypes. For example, treatment of *FMR1* knockout mice with monoacylglycerol lipase (MAGL) inhibitors to increase endocannabinoid signaling tone has been shown to normalize cortical responses to sound and diminish anxiety-like behaviors [42] and restore mGluR5-mediated long-term depression in brain slices taken from the ventral striatum of *FMR1* knockout mice [27]. Moreover, mice lacking the CB_1_ receptor display several changes in social behavior and communication both during early development and in adulthood, further supporting the role of the ECS in FXS- and ASD-like phenotypes [43]. Indeed, inhibition of the endocannabinoid producing enzyme, DAGL-α, induces ASD-like behavior and other co-morbid phenotypes in adult C57BL/J mice [44]. In humans, rare heterozygous genetic (missense) variants in *CNR1* and *DAGLA*, the genes encoding the CB_1_ receptor and the DAGL-α enzyme, have been shown to be associated with sleep and memory disorders—alone or in combination with anxiety, and with seizures and neurodevelopmental disorders, including abnormalities of behavior and brain morphology similar to those observed in FXS patients [45]. In contrast, rare missense variants in *MGLL*, *FAAH*, and *CNR2*, the genes encoding monoacylglycerol lipase, fatty acid amide hydrolase, and the CB_2_ receptor, respectively, were not associated with any abnormal neurological phenotypes in the patients examined in this study. Similarly, in clinical studies investigating gaze duration to facial stimuli, a behavior frequently altered in ASD and FXS patients, polymorphisms in the *CNR1* gene were shown to modulate striatal responses and gaze duration to happy faces [46, 47]. Together, these findings implicate the endocannabinoid-CB_1_ receptor signaling system in psychological and behavioral conditions involving altered responsivity to emotional and social stimuli such as those observed in FXS (for reviews, see references [48–50]).

### Cannabidiol effects on the CNS

**Fig. 3:**
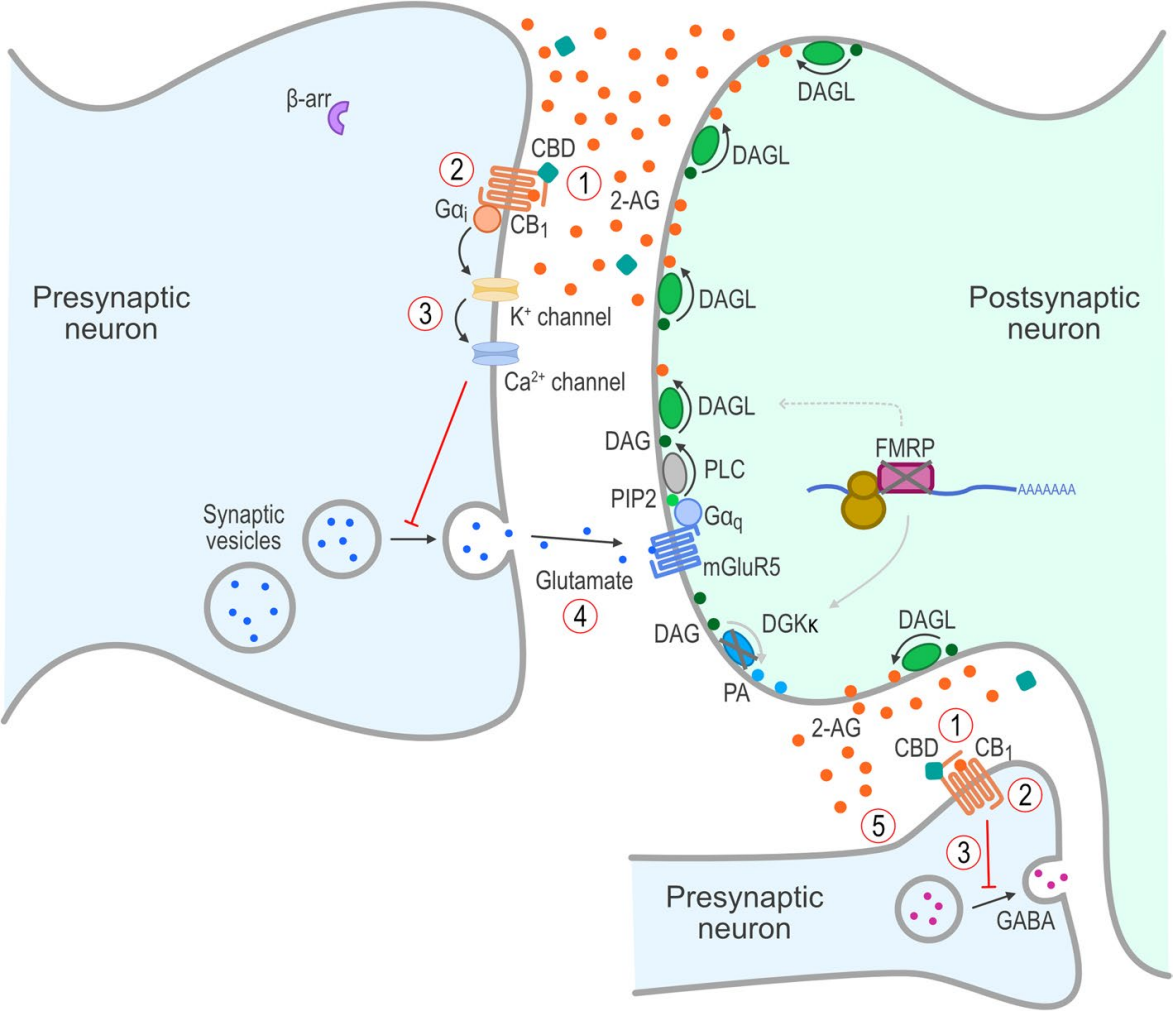
Proposed mechanism(s) of action of cannabidiol on the ECS in FXS. Treatment of FXS with cannabidiol is proposed to lead to (1) cannabidiol’s acting as a negative allosteric modulator (NAM) at the CB_1_ receptors, resulting in (2) reduction of β-arrestin recruitment, along with prevention of internalization and desensitization of CB_1_ receptors in the presence of ectopic/abnormal 2-AG, which leads to (3) restoration of retrograde inhibitory signaling and (4) reduction in glutamate release and activation of mGluR5 receptors and (5) restoration of GABAergic function. 2-AG, 2-arachidonoylglycerol; β-arr, β-arrestin; CB_1_, cannabinoid type 1 receptor; CBD, cannabidiol; DAG, diacylglycerol; DAGL, diacylglycerol lipase; DGKκ, diacylglycerol kinase-κ; ECS, endocannabinoid system; FMRP, *FMR1* protein; FXS, fragile X syndrome; G, G proteins; GABA, γ-aminobutyric acid; mGluR5, group I metabotropic glutamate receptor 5; PA, phosphatidic acid; PIP2, phosphatidylinositol-4,5-bisphosphate; PLC, phospholipase C

Cannabidiol, the main non-euphoric component of the cannabis plant, has a variety of effects on the ECS and has been studied in a variety of neurodevelopmental and neuropsychiatric disorders (for reviews, see references [51, 52]). Cannabidiol acts as a negative allosteric modulator of 2-AG at CB_1_, thereby attenuating 2-AG–mediated CB_1_ receptor activation, internalization, and desensitization [53, 54]. Moreover, cannabidiol may reduce CB_1_ receptor internalization even in the absence of 2-AG, thereby increasing the relative membrane expression of functional CB_1_ receptors [53–57]. In its activity as a negative allosteric modulator, cannabidiol does not compete with 2-AG binding to CB_1_, but rather shifts the dose response to the right and reduces the apparent potency of 2-AG signaling through the CB_1_ receptor [53]. Moreover, cannabidiol interacts with fatty acid-binding proteins (FABP) that transport AEA to fatty acid amide hydrolase (FAAH), and reduces transport and catabolic loss of AEA [54, 58–62]. Introduction of exogenous cannabidiol, therefore, is hypothesized to restore functional retrograde ECS signaling, thereby normalizing the ECS in the absence of FMRP (Fig. 3).

If the net effect of loss of FMRP is diminished cannabinoid signaling due to receptor desensitization as an adaptation to 2-AG overproduction [38], then treatment with MAGL inhibitors to increase endocannabinoid tone could overcome desensitization of the CB_1_ receptor and show therapeutic potential [27, 42]. However, this approach would also lead to further desensitization and internalization of CB_1_ and ultimately exacerbate the loss of retrograde signaling at the presynaptic terminal. In contrast, cannabidiol treatment may increase localization of functional CB_1_ receptors in the presynaptic membrane and shift the 2-AG dose response curve at the CB_1_ receptor to the right [53], diminishing CB_1_ receptor desensitization due to ectopic 2-AG release and enabling CB_1_ receptor function to contribute to synaptic plasticity. An interesting analogy is that desensitization and internalization of CB_1_ receptors in FXS is similar to hearing loss. Hearing loss can be overcome by increasing the volume of the sound over “normal” levels (similar to elevating 2-AG levels with MAGL inhibitors) but can cause further damage to the hairs (stereocilia) in the ears leading to further hearing loss and exacerbating the problem (similar to producing further CB_1_ receptor desensitization and internalization due to the increased endocannabinoid tone caused by treatment with MAGL-inhibitors). Cannabidiol by comparison, increases the levels of functional cannabinoid receptors at the plasma membrane, which could be viewed as restoring the responsivity of the hairs in the ear, and shifts the dose-response curve of 2-AG to the right (essentially lowering the background “noise”).

It is also important to note that there has been considerable preclinical and clinical interest in the role of group 1 metabotropic glutamate receptors in FXS (see [63] for review). In preclinical studies, *FMR1* knockout models in mice demonstrated that the absence of gene transcription and FMRP translation leads to increased protein synthesis at the postsynaptic membrane and (abnormal) enhancement of mGluR5 glutamatergic signaling and long-term depression, which are important components of synaptic plasticity, learning, and memory. Furthermore, administration of mGluR5 antagonists in *FMR1* knockout mice demonstrated a variety of benefits in this preclinical FXS model phenotype, including reduced seizures and anxiety-like behaviors [64]. However, clinical studies with mGluR5 antagonists have failed to show significant therapeutic utility [65]. While these clinical trials using mGluR5 antagonists have failed to show clinical utility in FXS patients, mGluR5 is also an integral component of the endocannabinoid signalosome. Specifically, the mGluR5 receptor is coupled to DAGL in the post-synaptic density, and upon stimulation by glutamate, it causes the liberation of 2-AG. This 2-AG then enables endocannabinoid-mediated retrograde signaling to the presynaptic CB_1_ receptor to produce long-term depression of glutamatergic transmission and other cellular signaling adaptations involved in neuronal plasticity, learning, and memory. In this scenario, mGluR5 antagonists could diminish excessive glutamatergic tone, as well as reduce whatever coupling of the mGluR5 receptor to endocannabinoid release is present and functional, but this would ultimately decrease endocannabinoid retrograde signaling and impede synaptic plasticity. It would have no effect on the background “noise” due to ectopic 2-AG release and would only diminish glutamate-induced 2-AG release.

### Cannabidiol effects on DNA methylation

As described earlier, the FMRP production is influenced by CGG repeat size and methylation [66]; however, the primary determinant is the degree to which *FMR1* alleles are methylated [15, 16]. The effect of a high degree of methylation differs in males and females: males with a hypermethylated FM generally do not produce FMRP, whereas females produce FMRP, with expression levels that correlate with the X-inactivation ratio of the affected allele. In females, inactivation of 1 of the 2 X chromosomes is a random process, potentially leading to differential intra and inter tissue patterns of FMRP expression. Furthermore, the normal, nonmutated X chromosome may also be affected by methylation and may produce less FMRP. The overall production of FMRP in females is determined by the extent of activation of the normal alleles.

Emerging evidence suggests that cannabidiol may regulate DNA methylation. Methionine synthesis is decreased by cannabidiol treatment [67]. Methionine serves as the substrate for methionine adenosyl transferase, which yields S-adenosylmethionine, which in turn is the key biochemical moiety involved in methyl group transfers to DNA through the action of DNA methyltransferases. This may lead to reduced DNA methylation. Cannabidiol was found to modulate DNA methylation in the prefrontal cortex and hippocampus of mice [68, 69]. In these pre-clinical studies, using the forced swim test model in mice, cannabidiol had an antidepressant-like effect and modulated DNA methylation in the prefrontal cortex and hippocampus, brain regions relevant for depression neurobiology [68]. Cannabidiol-treated mice showed a small skew toward global hypomethylation in hippocampal tissue [69]. In addition, genes for cell adhesion and migration, dendritic spine development, and excitatory postsynaptic potential were found to be enriched among the genes affected by cannabidiol-altered DNA methylation [69]. The effects of cannabidiol on DNA methylation in the FXS animal models have not been reported. These emerging results suggest that the DNA methylation epigenome may also be a key substrate for the long-term neurochemical and behavioral effects of cannabidiol.

### Other effects of cannabidiol potentially related to FXS

Several other effects of cannabidiol may provide therapeutic benefit in patients with FXS. Serotonin 5HT_1A_ receptors have been implicated in anxiety and depression, with most 5HT_1A_ receptor agonists exerting anxiolytic activity [70]. Cannabidiol binds to the 5HT_1A_ receptor with moderate affinity and possesses agonist efficacy in 5HT_1A_ signal transduction studies [71]. Cannabidiol has also been shown to act as a positive allosteric modulator at GABA_A_ receptors [72]. Cannabidiol’s ability to enhance endocannabinoid levels and facilitate GABAergic transmission may serve to improve the balance in inhibitory and excitatory transmission and help restore neuronal function and synaptic plasticity in patients with FXS. Cannabidiol is also a dopamine D_2_ partial agonist [73]. Moreover, cannabidiol interacts with dopamine D_3_ receptors [74] and reduces the expression of dopamine D_3_ receptors in a rat model of schizophrenia [75]. This is an area of active investigation and may indicate that cannabidiol has a fairly broad neuropharmacological mechanism of action.

### CONNECT-FX trial with transdermal cannabidiol gel

Because of the proposed role of dysregulation of the ECS in FXS, a signal-finding, open-label trial [33] and a randomized, double-blind, placebo-controlled trial have been conducted with ZYN002 in patients with FXS. ZYN002 is a pharmaceutically manufactured permeation-enhanced transdermal cannabidiol gel in development for the treatment of behavioral symptoms in FXS. The open-label trial found that ZYN002 was well tolerated and was associated with reduced anxiety and behavioral symptoms in children and adolescents with FXS [33]. The results from the open-label trial led to a phase 3 randomized controlled trial of ZYN002 in patients with FXS. CONNECT-FX is the largest controlled trial ever performed in FXS [76] and is described in more detail in the accompanying article in this journal [77]. In the intent-to-treat population, numerical improvements in Aberrant Behavior Checklist-Community FXS (ABC-C_FXS_) Social Avoidance, Irritability, and Socially Unresponsive/Lethargic subscale scores were greater in the ZYN002 group than in the placebo group; however, the differences were not statistically significant. A pre-planned ad hoc analysis, defined prior to breaking the study blind, was conducted to evaluate the efficacy of ZYN002 vs placebo in patients with ≥90% methylation of the promoter region of the *FMR1* gene. In patients with ≥90% methylation, ZYN002 was superior to placebo in multiple analyses. ZYN002 was associated with a statistically significant mean improvement from baseline in Social Avoidance vs placebo. In addition, the proportions of patients attaining a threshold of clinically meaningful within-patient change in Social Avoidance and Irritability were significantly greater with ZYN002 vs placebo. Moreover, there was a statistically significantly higher percentage of caregiver-reported improvements for Social Avoidance, Social Interaction, and Irritable Behaviors with ZYN002 vs placebo. ZYN002 was also found to be well tolerated in this study. A post hoc analysis indicated that the treatment effect of ZYN002 in improvement of Social Avoidance was most pronounced in patients who had 100% methylation of their *FMR1* gene promoter, thereby supporting the idea that ZYN002 is most effective in patients with complete silencing of the *FMR1* gene. Thus, the results of the CONNECT-FX trial are consistent with the proposed mechanisms of action of cannabidiol in FXS described in this article.

### Future directions

Much of the research on the roles of the ECS in FXS has been conducted in the past decade and is rapidly developing. The mechanisms discussed in this review are based largely on data obtained from animal models, which are amenable to experimental research, but which may not always accurately reflect the human disease process (e.g., the negative results obtained with mGluR5 antagonists in clinical studies in FXS). One area of preclinical research that may provide important insights is assessing the relative contributions of the effects of cannabidiol on the various implicated signaling pathways, such as CB_1_ receptor signaling, DNA methylation, serotonin 5HT_1A_ signal transduction, GABA receptor signaling, and dopamine D_2_ and D_3_ receptor signaling. In clinical research, it would be beneficial to have more detailed assessments of the effects of acute and chronic administration of cannabidiol on specific regions of the brain [78, 79]. There is also a need for additional controlled clinical trials of cannabidiol in patients with neurodevelopmental disorders such as FXS and ASD. In particular, it will be important to identify appropriate target populations in FXS and ASD that may benefit most from cannabidiol treatment.

## Conclusions

FXS is caused by deficiency or absence of FMRP, typically due to the presence of >200 CGG repeats and methylation in the promoter region of the *FMR1* gene. The absence of FMRP downregulates the ECS signaling, which has been implicated in FXS pathogenesis. Synaptic homeostasis and plasticity may be regulated by the ECS through the postsynaptic “on demand” production of endocannabinoids, which then bind to CB_1_ receptors on presynaptic terminals, resulting in regulation of glutamate signaling and GABAergic signaling. The ECS-mediated feedback inhibition and synaptic plasticity are thought to be disrupted in FXS due to dysregulation of enzymes that are integral to the ECS (e.g., DAGL), leading to overstimulation, desensitization, and internalization of presynaptic CB_1_ receptors. Cannabidiol may help restore synaptic homeostasis by acting as a negative allosteric modulator of CB_1_, thereby attenuating CB_1_ receptor overstimulation, internalization, and desensitization. Moreover, cannabidiol has effects on DNA methylation, 5HT_1A_ signal transduction, GABA_A_ receptor signaling, and dopamine D_2_ and D_3_ receptor signaling, which may contribute to beneficial effects in patients with FXS. Consistent with these proposed mechanisms of action of cannabidiol in FXS, the transdermal cannabidiol gel, ZYN002, was associated with improvements in measures of social avoidance, irritability, and social interaction in the CONNECT-FX trial, particularly among patients with ≥90% methylation of the *FMR1* gene.

## Data Availability

Not applicable.

## Abbreviations

2-AG: 2-Arachidonoylglycerol
ABC-C_FXS_: Aberrant Behavior Checklist-Community Fragile X Syndrome
AEA: Anandamide
ASD: Autism spectrum disorder
CB1: Cannabinoid receptor 1
CB2: Cannabinoid receptor 2
CGG: Cytosine, guanine, guanine
CNS: Central nervous system
DAGL: Diacylglycerol lipase
DGKκ: Diacylglycerol kinase-κ
ECS: Endocannabinoid system
FAAH: Fatty acid amide hydrolase
FABP: Fatty acid-binding protein
FM: Full mutation
FXS: Fragile X syndrome
GABA: Gamma-aminobutyric acid
MAGL: Monoacylglycerol lipase
mGluR5: Metabotropic glutamate receptor 5
mRNA: Messenger RNA
